# 1-Ethyl­sulfinyl-2-(4-iodo­phen­yl)naphtho­[2,1-*b*]furan

**DOI:** 10.1107/S1600536810029144

**Published:** 2010-07-31

**Authors:** Hong Dae Choi, Pil Ja Seo, Byeng Wha Son, Uk Lee

**Affiliations:** a Department of Chemistry, Dongeui University, San 24 Kaya-dong Busanjin-gu, Busan 614-714, Republic of Korea; b Department of Chemistry, Pukyong National University, 599-1 Daeyeon 3-dong, Nam-gu, Busan 608-737, Republic of Korea

## Abstract

In the title compound, C_20_H_15_IO_2_S, the 4-iodo­phenyl ring makes a dihedral angle of 44.21 (7)° with the plane of the naphtho­furan fragment. In the crystal, mol­ecules are linked by weak inter­molecular C—H⋯O and C—H⋯π inter­actions.

## Related literature

For the pharmacological activity of naphtho­furan compounds, see: Einhorn *et al.* (1984 ▶); Hranjec *et al.* (2003 ▶); Mahadevan & Vaidya (2003 ▶). For the structures of related 2-aryl-1-(methyl­sulfin­yl)naphtho­[2,1-*b*]furan derivatives, see: Choi *et al.* (2006 ▶, 2010 ▶).
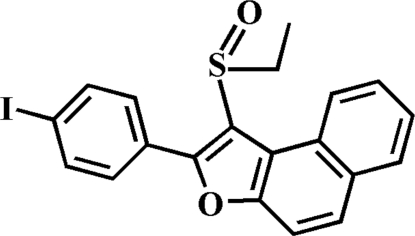


## Experimental

### 

#### Crystal data

C_20_H_15_IO_2_S
*M* *_r_* = 446.28
Monoclinic, 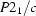

*a* = 9.1240 (5) Å
*b* = 12.4302 (6) Å
*c* = 15.8520 (8) Å
β = 105.899 (2)°
*V* = 1729.05 (15) Å^3^
*Z* = 4
Mo *K*α radiation
μ = 1.98 mm^−1^
*T* = 174 K
0.27 × 0.23 × 0.10 mm

#### Data collection

Bruker SMART APEXII CCD diffractometer
Absorption correction: multi-scan (*SADABS*; Bruker, 2009 ▶) *T*_min_ = 0.619, *T*_max_ = 0.825
14865 measured reflections
3937 independent reflections
3511 reflections with *I* > 2σ(*I*)
*R*_int_ = 0.038

#### Refinement

*R*[*F* ^2^ > 2σ(*F* ^2^)] = 0.028
*wR*(*F* ^2^) = 0.084
*S* = 1.09
3937 reflections
218 parameters
H-atom parameters constrained
Δρ_max_ = 0.37 e Å^−3^
Δρ_min_ = −1.28 e Å^−3^

### 

Data collection: *APEX2* (Bruker, 2009 ▶); cell refinement: *SAINT* (Bruker, 2009 ▶); data reduction: *SAINT*; program(s) used to solve structure: *SHELXS97* (Sheldrick, 2008 ▶); program(s) used to refine structure: *SHELXL97* (Sheldrick, 2008 ▶); molecular graphics: *ORTEP-3* (Farrugia, 1997 ▶) and *DIAMOND* (Brandenburg, 1998 ▶); software used to prepare material for publication: *SHELXL97*.

## Supplementary Material

Crystal structure: contains datablocks I. DOI: 10.1107/S1600536810029144/ds2042sup1.cif

Structure factors: contains datablocks I. DOI: 10.1107/S1600536810029144/ds2042Isup2.hkl

Additional supplementary materials:  crystallographic information; 3D view; checkCIF report

## Figures and Tables

**Table 1:** Hydrogen-bond geometry (Å, °) *Cg* is the centroid of the C13–C18 4-iodo­phenyl ring.

*D*—H⋯*A*	*D*—H	H⋯*A*	*D*⋯*A*	*D*—H⋯*A*
C20—H20*C*⋯O2^i^	0.98	2.60	3.454 (4)	145
C19—H19*B*⋯*Cg*^ii^	0.99	2.77	3.501 (3)	131

Symmetry codes: (i) 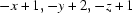; (ii) 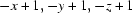.

**Fig. 1.**
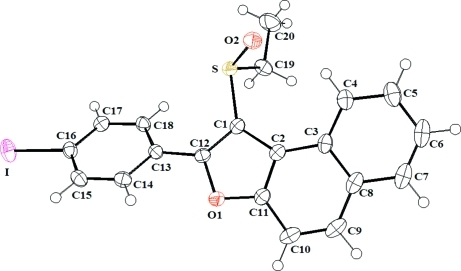
The molecular structure of the title compound with the atom numbering scheme. Displacement ellipsoids are drawn at the 50% probability level. H atoms are presented as a small spheres of arbitrary radius.

**Fig. 2.**
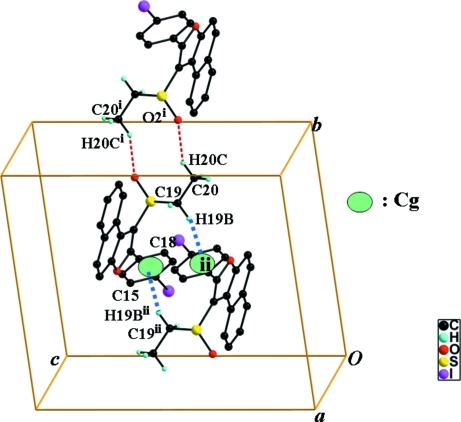
C—H···O and C—H···π interactions (dotted lines) in the crystal structure of the title compound. Cg denotes the ring centroid. [Symmetry codes: ?? (i) - x + 1, - y + 2, - z + 1; (ii) - x + 1, - y + 1, - z +1.]

## supplementary crystallographic information

### Comment

Compounds containing a naphthofuran moiety show diverse pharmacological properties such as antibacterial, antitumor and anthelmintic activities (Einhorn *et al.*, 1984, Hranjec *et al.*, 2003, Mahadevan & Vaidya, 2003). As a part of our ongoing studies of the substituent effect on the solid state structures of 2-aryl-1-(methylsulfinyl)naphtho[2,1-b]furan analogues (Choi *et al.*, 2006, 2010), we report the crystal structure of the title compound (Fig. 1).

The naphthofuran unit is essentially planar, with a mean deviation of 0.044 (2) Å from the least-squares plane defined by the thirteen constituent atoms. The dihedral angle formed by the naphthofuran plane and the 4-iodophenyl ring is 44.21 (7)°. The crystal packing (Fig. 2) is stabilized by a weak intermolecular C—H···O hydrogen bond between the methyl H atom of the ethyl group and the oxygen of the S═O unit, with a C20—H20C···O2^i^ (Table 1). The molecular packing (Fig. 2) is further stabilized by an intermolecular C—H···π interaction between the methylene H atom of the ethyl group and the 4-iodophenyl ring of an adjacent molecule, with a C19—H19B···Cg^ii^ (Table 1; Cg is the centroid of the C13–C18 4-iodophenyl ring).

### Experimental

77% 3-chloroperoxybenzoic acid (157 mg, 0.7 mmol) was added in small portions to a stirred solution of 1-ethylsulfanyl-2-(4-iodophenyl)naphtho [2,1-b]furan (301 mg, 0.7 mmol) in dichloromethane (30 mL) at 273 K. After being stirred at room temperature for 5h, the mixture was washed with saturated sodium bicarbonate solution and the organic layer was separated, dried over magnesium sulfate, filtered and concentrated at reduced pressure. The residue was purified by column chromatography (hexane–ethyl acetate, 2:1 v/v) to afford the title compound as a colorless solid [yield 74%, m.p. 440–441 K; *R*_f_ = 0.53 (hexane–ethyl acetate, 2:1 v/v)]. Single crystals suitable for X-ray diffraction were prepared by slow evaporation of a solution of the title compound in acetone at room temperature.

### Refinement

All H atoms were positioned geometrically and refined using a riding model, with C—H = 0.95 Å for aryl, 0.99 Å for methylene, and 0.98 Å for methyl H atoms. *U*_iso_(H) = 1.2*U*_eq_(C) for aryl and methylene H atoms, and 1.5*U*_eq_(C) for methyl H atoms.

### Crystal data

**Table:**

C_20_H_15_IO_2_S	*F*(000) = 880
*M**_r_* = 446.28	*D*_x_ = 1.714 Mg m^−^^3^
Monoclinic, *P*2_1_/*c*	Mo *K*α radiation, λ = 0.71073 Å
*a* = 9.1240 (5) Å	Cell parameters from 3937 reflections
*b* = 12.4302 (6) Å	θ = 2.1–27.5°
*c* = 15.8520 (8) Å	µ = 1.98 mm^−^^1^
β = 105.899 (2)°	*T* = 174 K
*V* = 1729.05 (15) Å^3^	Block, colourless
*Z* = 4	0.27 × 0.23 × 0.10 mm

### Data collection

**Table:**

Bruker SMART APEXII CCD diffractometer	3937 independent reflections
Radiation source: rotating anode	3511 reflections with *I* > 2σ(*I*)
graphite multilayer	*R*_int_ = 0.038
Detector resolution: 10.0 pixels mm^-1^	θ_max_ = 27.5°, θ_min_ = 2.1°
φ and ω scans	*h* = −11→11
Absorption correction: multi-scan (*SADABS*; Bruker, 2009)	*k* = −16→13
*T*_min_ = 0.619, *T*_max_ = 0.825	*l* = −20→19
14865 measured reflections	

### Refinement

**Table:**

Refinement on *F*^2^	Primary atom site location: structure-invariant direct methods
Least-squares matrix: full	Secondary atom site location: difference Fourier map
*R*[*F*^2^ > 2σ(*F*^2^)] = 0.028	Hydrogen site location: difference Fourier map
*wR*(*F*^2^) = 0.084	H-atom parameters constrained
*S* = 1.09	*w* = 1/[σ^2^(*F*_o_^2^) + (0.0453*P*)^2^ + 0.6527*P*] where *P* = (*F*_o_^2^ + 2*F*_c_^2^)/3
3937 reflections	(Δ/σ)_max_ = 0.001
218 parameters	Δρ_max_ = 0.37 e Å^−^^3^
0 restraints	Δρ_min_ = −1.28 e Å^−^^3^

### Special details

**Table:**

Geometry. All esds (except the esd in the dihedral angle between two l.s. planes) are estimated using the full covariance matrix. The cell esds are taken into account individually in the estimation of esds in distances, angles and torsion angles; correlations between esds in cell parameters are only used when they are defined by crystal symmetry. An approximate (isotropic) treatment of cell esds is used for estimating esds involving l.s. planes.
Refinement. Refinement of F^2^ against ALL reflections. The weighted R-factor wR and goodness of fit S are based on F^2^, conventional R-factors R are based on F, with F set to zero for negative F^2^. The threshold expression of F^2^ > 2sigma(F^2^) is used only for calculating R-factors(gt) etc. and is not relevant to the choice of reflections for refinement. R-factors based on F^2^ are statistically about twice as large as those based on F, and R- factors based on ALL data will be even larger.

### Fractional atomic coordinates and isotropic or equivalent isotropic displacement parameters (Å^2^)

**Table:**

	*x*	*y*	*z*	*U*_iso_*/*U*_eq_	
I	0.00380 (2)	0.277333 (14)	0.601089 (11)	0.03776 (9)	
S	0.45899 (6)	0.76556 (4)	0.55355 (4)	0.02160 (13)	
O1	0.70925 (17)	0.52550 (13)	0.67638 (10)	0.0239 (3)	
O2	0.48599 (18)	0.87424 (14)	0.59467 (10)	0.0285 (4)	
C1	0.6068 (2)	0.67815 (18)	0.60936 (13)	0.0205 (4)	
C2	0.7714 (2)	0.69096 (19)	0.63592 (13)	0.0213 (4)	
C3	0.8780 (3)	0.77275 (18)	0.62844 (15)	0.0238 (5)	
C4	0.8389 (3)	0.8777 (2)	0.59683 (15)	0.0275 (5)	
H4	0.7348	0.8988	0.5798	0.033*	
C5	0.9491 (3)	0.9498 (2)	0.59032 (17)	0.0364 (6)	
H5	0.9206	1.0196	0.5674	0.044*	
C6	1.1048 (3)	0.9209 (3)	0.61747 (18)	0.0406 (7)	
H6	1.1807	0.9712	0.6128	0.049*	
C7	1.1455 (3)	0.8211 (3)	0.65028 (16)	0.0359 (6)	
H7	1.2505	0.8026	0.6688	0.043*	
C8	1.0358 (3)	0.7438 (2)	0.65766 (15)	0.0289 (5)	
C9	1.0814 (3)	0.6397 (2)	0.69298 (15)	0.0311 (5)	
H9	1.1868	0.6221	0.7095	0.037*	
C10	0.9800 (3)	0.5649 (2)	0.70387 (15)	0.0292 (5)	
H10	1.0113	0.4967	0.7297	0.035*	
C11	0.8256 (2)	0.59444 (19)	0.67454 (14)	0.0230 (4)	
C12	0.5766 (2)	0.57813 (18)	0.63581 (13)	0.0206 (4)	
C13	0.4390 (2)	0.51475 (17)	0.62874 (13)	0.0203 (4)	
C14	0.4302 (3)	0.44819 (18)	0.69846 (14)	0.0242 (5)	
H14	0.5106	0.4485	0.7512	0.029*	
C15	0.3056 (3)	0.38198 (19)	0.69132 (15)	0.0259 (5)	
H15	0.3000	0.3372	0.7389	0.031*	
C16	0.1890 (3)	0.38172 (18)	0.61392 (14)	0.0234 (4)	
C17	0.1941 (2)	0.44740 (18)	0.54422 (14)	0.0234 (4)	
H17	0.1127	0.4469	0.4918	0.028*	
C18	0.3187 (2)	0.51365 (18)	0.55162 (14)	0.0225 (4)	
H18	0.3227	0.5589	0.5040	0.027*	
C19	0.5111 (3)	0.77015 (18)	0.45098 (15)	0.0274 (5)	
H19A	0.6212	0.7860	0.4630	0.033*	
H19B	0.4914	0.6992	0.4216	0.033*	
C20	0.4199 (4)	0.8559 (2)	0.39126 (19)	0.0442 (7)	
H20A	0.3111	0.8389	0.3779	0.066*	
H20B	0.4506	0.8585	0.3367	0.066*	
H20C	0.4391	0.9260	0.4206	0.066*	

### Atomic displacement parameters (Å^2^)

**Table:**

	*U*^11^	*U*^22^	*U*^33^	*U*^12^	*U*^13^	*U*^23^
I	0.04151 (13)	0.03477 (14)	0.03818 (12)	−0.01862 (7)	0.01291 (8)	−0.00299 (6)
S	0.0202 (3)	0.0193 (3)	0.0249 (3)	0.0019 (2)	0.0054 (2)	−0.0001 (2)
O1	0.0217 (7)	0.0252 (8)	0.0241 (8)	0.0026 (6)	0.0052 (6)	0.0041 (6)
O2	0.0315 (9)	0.0215 (9)	0.0344 (9)	0.0020 (7)	0.0120 (7)	−0.0055 (6)
C1	0.0198 (10)	0.0248 (11)	0.0174 (9)	0.0003 (9)	0.0061 (7)	−0.0011 (8)
C2	0.0201 (10)	0.0270 (11)	0.0173 (10)	0.0010 (9)	0.0059 (7)	−0.0020 (8)
C3	0.0231 (11)	0.0301 (13)	0.0204 (10)	−0.0055 (9)	0.0097 (8)	−0.0048 (9)
C4	0.0302 (11)	0.0277 (12)	0.0269 (11)	−0.0060 (10)	0.0118 (9)	−0.0060 (9)
C5	0.0441 (15)	0.0345 (15)	0.0338 (13)	−0.0145 (12)	0.0158 (11)	−0.0081 (11)
C6	0.0355 (14)	0.0529 (18)	0.0376 (14)	−0.0217 (13)	0.0169 (11)	−0.0139 (13)
C7	0.0257 (12)	0.0539 (18)	0.0304 (13)	−0.0126 (12)	0.0114 (9)	−0.0121 (12)
C8	0.0221 (10)	0.0446 (14)	0.0212 (11)	−0.0045 (10)	0.0077 (8)	−0.0077 (10)
C9	0.0195 (10)	0.0498 (16)	0.0232 (11)	0.0065 (11)	0.0045 (8)	−0.0061 (11)
C10	0.0234 (11)	0.0410 (14)	0.0221 (11)	0.0094 (10)	0.0044 (8)	−0.0018 (10)
C11	0.0206 (10)	0.0285 (12)	0.0201 (10)	0.0004 (9)	0.0060 (7)	−0.0013 (8)
C12	0.0205 (10)	0.0217 (11)	0.0193 (10)	0.0020 (8)	0.0048 (7)	−0.0007 (8)
C13	0.0235 (10)	0.0186 (10)	0.0203 (10)	0.0004 (8)	0.0085 (8)	−0.0001 (8)
C14	0.0264 (11)	0.0254 (12)	0.0198 (10)	0.0012 (9)	0.0045 (8)	0.0012 (8)
C15	0.0330 (12)	0.0230 (12)	0.0232 (11)	−0.0001 (9)	0.0105 (9)	0.0035 (9)
C16	0.0264 (11)	0.0195 (11)	0.0265 (11)	−0.0031 (9)	0.0110 (8)	−0.0029 (9)
C17	0.0237 (10)	0.0262 (11)	0.0205 (10)	0.0012 (9)	0.0065 (8)	−0.0024 (9)
C18	0.0261 (10)	0.0236 (11)	0.0191 (10)	0.0006 (9)	0.0083 (8)	0.0029 (8)
C19	0.0360 (13)	0.0250 (13)	0.0207 (11)	0.0006 (10)	0.0067 (9)	0.0010 (9)
C20	0.0500 (17)	0.0410 (16)	0.0373 (15)	0.0042 (14)	0.0045 (12)	0.0157 (12)

### Geometric parameters (Å, °)

**Table:**

I—C16	2.096 (2)	C9—C10	1.356 (4)
S—O2	1.4908 (18)	C9—H9	0.9500
S—C1	1.769 (2)	C10—C11	1.406 (3)
S—C19	1.816 (3)	C10—H10	0.9500
O1—C11	1.371 (3)	C12—C13	1.460 (3)
O1—C12	1.371 (2)	C13—C14	1.401 (3)
C1—C12	1.364 (3)	C13—C18	1.402 (3)
C1—C2	1.453 (3)	C14—C15	1.382 (3)
C2—C11	1.376 (3)	C14—H14	0.9500
C2—C3	1.434 (3)	C15—C16	1.386 (3)
C3—C4	1.407 (3)	C15—H15	0.9500
C3—C8	1.432 (3)	C16—C17	1.385 (3)
C4—C5	1.372 (4)	C17—C18	1.382 (3)
C4—H4	0.9500	C17—H17	0.9500
C5—C6	1.413 (4)	C18—H18	0.9500
C5—H5	0.9500	C19—C20	1.515 (3)
C6—C7	1.358 (4)	C19—H19A	0.9900
C6—H6	0.9500	C19—H19B	0.9900
C7—C8	1.414 (4)	C20—H20A	0.9800
C7—H7	0.9500	C20—H20B	0.9800
C8—C9	1.426 (4)	C20—H20C	0.9800
			
O2—S—C1	109.02 (10)	O1—C11—C10	122.8 (2)
O2—S—C19	108.10 (10)	C2—C11—C10	125.5 (2)
C1—S—C19	96.68 (11)	C1—C12—O1	110.63 (18)
C11—O1—C12	106.32 (17)	C1—C12—C13	135.2 (2)
C12—C1—C2	106.90 (19)	O1—C12—C13	114.11 (18)
C12—C1—S	121.54 (16)	C14—C13—C18	118.8 (2)
C2—C1—S	131.56 (17)	C14—C13—C12	119.50 (18)
C11—C2—C3	119.1 (2)	C18—C13—C12	121.60 (19)
C11—C2—C1	104.5 (2)	C15—C14—C13	120.7 (2)
C3—C2—C1	136.4 (2)	C15—C14—H14	119.6
C4—C3—C8	118.8 (2)	C13—C14—H14	119.6
C4—C3—C2	125.1 (2)	C14—C15—C16	119.2 (2)
C8—C3—C2	116.1 (2)	C14—C15—H15	120.4
C5—C4—C3	120.8 (2)	C16—C15—H15	120.4
C5—C4—H4	119.6	C17—C16—C15	121.3 (2)
C3—C4—H4	119.6	C17—C16—I	119.33 (16)
C4—C5—C6	120.5 (3)	C15—C16—I	119.35 (17)
C4—C5—H5	119.7	C18—C17—C16	119.3 (2)
C6—C5—H5	119.7	C18—C17—H17	120.3
C7—C6—C5	119.7 (2)	C16—C17—H17	120.3
C7—C6—H6	120.2	C17—C18—C13	120.6 (2)
C5—C6—H6	120.2	C17—C18—H18	119.7
C6—C7—C8	121.8 (3)	C13—C18—H18	119.7
C6—C7—H7	119.1	C20—C19—S	110.37 (19)
C8—C7—H7	119.1	C20—C19—H19A	109.6
C7—C8—C9	120.8 (2)	S—C19—H19A	109.6
C7—C8—C3	118.3 (3)	C20—C19—H19B	109.6
C9—C8—C3	120.9 (2)	S—C19—H19B	109.6
C10—C9—C8	122.4 (2)	H19A—C19—H19B	108.1
C10—C9—H9	118.8	C19—C20—H20A	109.5
C8—C9—H9	118.8	C19—C20—H20B	109.5
C9—C10—C11	115.8 (2)	H20A—C20—H20B	109.5
C9—C10—H10	122.1	C19—C20—H20C	109.5
C11—C10—H10	122.1	H20A—C20—H20C	109.5
O1—C11—C2	111.62 (18)	H20B—C20—H20C	109.5
			
O2—S—C1—C12	132.48 (18)	C3—C2—C11—O1	−179.95 (19)
C19—S—C1—C12	−115.73 (19)	C1—C2—C11—O1	−1.9 (2)
O2—S—C1—C2	−47.7 (2)	C3—C2—C11—C10	−3.9 (3)
C19—S—C1—C2	64.1 (2)	C1—C2—C11—C10	174.1 (2)
C12—C1—C2—C11	1.5 (2)	C9—C10—C11—O1	176.2 (2)
S—C1—C2—C11	−178.31 (17)	C9—C10—C11—C2	0.6 (3)
C12—C1—C2—C3	179.1 (2)	C2—C1—C12—O1	−0.7 (2)
S—C1—C2—C3	−0.8 (4)	S—C1—C12—O1	179.19 (14)
C11—C2—C3—C4	−174.8 (2)	C2—C1—C12—C13	−178.5 (2)
C1—C2—C3—C4	7.9 (4)	S—C1—C12—C13	1.3 (4)
C11—C2—C3—C8	3.8 (3)	C11—O1—C12—C1	−0.5 (2)
C1—C2—C3—C8	−173.5 (2)	C11—O1—C12—C13	177.89 (17)
C8—C3—C4—C5	2.9 (3)	C1—C12—C13—C14	−143.3 (3)
C2—C3—C4—C5	−178.6 (2)	O1—C12—C13—C14	38.9 (3)
C3—C4—C5—C6	−1.7 (4)	C1—C12—C13—C18	40.6 (4)
C4—C5—C6—C7	0.0 (4)	O1—C12—C13—C18	−137.2 (2)
C5—C6—C7—C8	0.5 (4)	C18—C13—C14—C15	0.4 (3)
C6—C7—C8—C9	−179.5 (2)	C12—C13—C14—C15	−175.7 (2)
C6—C7—C8—C3	0.7 (4)	C13—C14—C15—C16	0.2 (4)
C4—C3—C8—C7	−2.4 (3)	C14—C15—C16—C17	−0.9 (4)
C2—C3—C8—C7	178.9 (2)	C14—C15—C16—I	177.76 (17)
C4—C3—C8—C9	177.9 (2)	C15—C16—C17—C18	0.7 (3)
C2—C3—C8—C9	−0.8 (3)	I—C16—C17—C18	−177.86 (17)
C7—C8—C9—C10	177.7 (2)	C16—C17—C18—C13	0.0 (3)
C3—C8—C9—C10	−2.6 (4)	C14—C13—C18—C17	−0.6 (3)
C8—C9—C10—C11	2.7 (3)	C12—C13—C18—C17	175.5 (2)
C12—O1—C11—C2	1.5 (2)	O2—S—C19—C20	−57.7 (2)
C12—O1—C11—C10	−174.6 (2)	C1—S—C19—C20	−170.25 (19)

### Hydrogen-bond geometry (Å, °)

**Table:**

Cg is the centroid of the C13–C18 4-iodophenyl ring.

**Table:**

*D*—H···*A*	*D*—H	H···*A*	*D*···*A*	*D*—H···*A*
C20—H20C···O2^i^	0.98	2.60	3.454 (4)	145
C19—H19B···Cg^ii^	0.99	2.77	3.501 (3)	131

Symmetry codes: (i) −*x*+1, −*y*+2, −*z*+1; (ii) −*x*+1, −*y*+1, −*z*+1.
